# Expanding the Chondroitin Sulfate Glycoproteome — But How Far?

**DOI:** 10.3389/fcell.2021.695970

**Published:** 2021-08-13

**Authors:** Fredrik Noborn, Mahnaz Nikpour, Andrea Persson, Jonas Nilsson, Göran Larson

**Affiliations:** ^1^Department of Laboratory Medicine, Institute of Biomedicine, Sahlgrenska Academy at the University of Gothenburg, Gothenburg, Sweden; ^2^Proteomics Core Facility, Sahlgrenska Academy at the University of Gothenburg, Gothenburg, Sweden

**Keywords:** proteoglycans, glycosaminoglycans, chondroitin sulfate, core proteins, glycoproteomics, attachment site

## Abstract

Chondroitin sulfate proteoglycans (CSPGs) are found at cell surfaces and in connective tissues, where they interact with a multitude of proteins involved in various pathophysiological processes. From a methodological perspective, the identification of CSPGs is challenging, as the identification requires the combined sequencing of specific core proteins, together with the characterization of the CS polysaccharide modification(s). According to the current notion of CSPGs, they are often considered in relation to a functional role in which a given proteoglycan regulates a specific function in cellular physiology. Recent advances in glycoproteomic methods have, however, enabled the identification of numerous novel chondroitin sulfate core proteins, and their glycosaminoglycan attachment sites, in humans and in various animal models. In addition, these methods have revealed unexpected structural complexity even in the linkage regions. These findings indicate that the number and structural complexity of CSPGs are much greater than previously perceived. In light of these findings, the prospect of finding additional CSPGs, using improved methods for structural and functional characterizations, and studying novel sample matrices in humans and in animal models is discussed. Further, as many of the novel CSPGs are found in low abundance and with not yet assigned functions, these findings may challenge the traditional notion of defining proteoglycans. Therefore, the concept of proteoglycans is considered, discussing whether “a proteoglycan” should be defined mainly on the basis of an assigned function or on the structural evidence of its existence.

## Introduction

The concept of chondroitin sulfate proteoglycans (CSPGs) as discrete molecular entities first emerged during the late 1950s. At that time, chondroitin sulfate (CS) and protein complexes had been identified in hyaline cartilage, but the nature of the complexes remained elusive, and it was unclear whether the CS to protein association involved a covalent bond or not (Shatton and Schubert, 1954). In a pioneering study by Helen Muir, she showed that the CS chains were indeed covalently linked to the protein counterpart (Muir, 1958). A few years later, it was found that the CS polysaccharides are attached to serine residues of the core proteins via a tetrasaccharide linkage region, composed of Glucuronic acid (GlcA)—Galactose (Gal)—Galactose (Gal)—Xylose (Xyl) (Lindahl and Roden, 1966; Roden and Smith, 1966). Since then, an increasing number of different CSPGs have been identified, each distinguished by the primary sequence of the core protein and with different numbers of CS chains attached (Olson et al., 2006; Iozzo and Schaefer, 2015; Pomin and Mulloy, 2018; Toledo et al., 2020).

CSPGs are important components in connective tissue and fine-tunes a wide range of cellular processes, including neural development, growth factor signaling and inflammation (Hatano and Watanabe, 2020; Hussein et al., 2020). However, the structural identification of CSPGs is often difficult, as the identification requires the combined sequencing of specific core proteins together with the structural verification of any potential CS polysaccharides. Consequently, studies on identifying novel CSPGs, using earlier established biochemical techniques, were mostly focused on the characterization of single core proteins in a defined cellular or physiological context (Krusius and Ruoslahti, 1986; Fisher et al., 1989). Such techniques are typically based on different read out assays following enzymatic depolymerization with bacterial lyases and/or site directed mutagenesis of cloned proteins.

The lack of suitable analytical methods for large-scale analyses of CSPGs in biological samples has for a long time limited the ability to identify both novel CSPGs and assess the degree of heterogeneity of the CS glycoproteome across various systems.

This mini review will specifically focus on the recent advances in glycoproteomics to identify and characterize CSPGs in complex sample mixtures in humans and animal model systems. These methods include trypsin digestion, enrichment of acidic glycopeptides by strong anion-exchange chromatography (SAX), and incubation with chondroitinase ABC to reduce the length and complexity of the CS chains. The samples are then analyzed with reversed phase nano-liquid chromatography-tandem mass spectrometry (nLC-MS/MS) and evaluated by glycopeptide search algorithms, resulting in the discovery and characterization of several novel CSPGs both in vertebrates and invertebrates. In light of these findings, the structural and conceptual insights that can be provided by such attachment site-specific analysis of the CS glycoproteome are discussed. Further, as many of the novel CSPGs are found in low abundance and with no yet assigned functions, these findings may challenge the traditional notion of proteoglycans, in which the described proteoglycans often have an assigned function. Finally, the concept of the CS glycoproteome is discussed in relation to whether the glycoproteomic space should be regarded as a static or a dynamic entity.

## A Glycoproteomic Approach to Identify Novel Proteoglycans

In glycobiology, mass spectrometry-based strategies for investigating protein glycosylation (glycoproteomics) have become an increasingly important tool (Nilsson et al., 2009; Thaysen-Andersen et al., 2016; Narimatsu et al., 2018). Such strategies, well-covered in excellent reviews, are typically based on enrichment of glycopeptides and subsequent analysis with nLC-MS/MS to provide site-specific information of *N*- and *O*-glycans (Nilsson, 2016; Darula and Medzihradszky, 2018; Madsen et al., 2020; Chernykh et al., 2021). To further develop this concept, we established a glycoproteomic protocol for global characterization of CS-glycopeptides in human urine and cerebrospinal fluid (CSF) (Noborn et al., 2015; Noborn et al., 2021). At first, bikunin (also known as protein AMBP) was used as a model CSPG since it was relatively well characterized, available in large quantities from human urine and also used in some countries as a pharmaceutical agent to treat acute pancreatitis (Ly et al., 2011; Lord et al., 2020). Human bikunin has a single CS chain of 27–39 monosaccharides attached to the N-terminal end (Ser-10) of the core protein (Lord et al., 2013). We incubated pharmaceutical grade bikunin with chondroitinase ABC that generated free disaccharides and a residual hexasaccharide structure still attached to the core protein. Digestion with trypsin generated a defined CS-glycopeptide suitable in size for nLC-MS/MS analysis. The analysis enabled the identification of several specific glycosidic and bikunin peptide fragments, serving as a proof-of-concept that site-specific analysis of CSPGs is indeed a feasible strategy. We then enriched trypsin-digested CSPGs from human urine and CSF and incubated the enriched samples with chondroitinase ABC, and thereafter analyzed the resulting CS-glycopeptides by nLC-MS/MS. Generated data were evaluated through proteomic software with adjustments to allow for glycopeptide identification, enabling the identification of 13 novel human CSPGs in addition to 13 already established CSPGs. In Tables 1A,B, the novel proteoglycans identified in humans and different model systems through glycoproteomics are shown. Interestingly, several of the novel human CSPGs were traditionally defined as prohormones, which was surprising, as prohormones are not typically regarded to belong to the proteoglycan superfamily (Noborn et al., 2015). This approach demonstrates the structural and conceptual insights that can be provided by global attachment site-specific analysis of the CS glycoproteome.

**TABLE 1:** **A.** Novel human proteoglycans identified through glycoproteomics.

*Human*				

Protein name	Uniprot ID	Modification^*a*^	Site(s)^*b*^	References
Brain-specific angiogenesis inhibitor 2	O60241	CS	266*	Noborn et al., 2015
CD99L2 protein	H0Y4H3	CS	141*	Noborn et al., 2015
Cholecystokinin	P06307	CS	31	Noborn et al., 2015
Collagen and calcium-binding EGF domain containing protein 1	Q6UXH8	CS	385	Noborn et al., 2015
Dermcidin	P81605	CS	30*	Noborn et al., 2015
Laminin subunit alpha-4	Q16363	CS	40*	Noborn et al., 2015
Laminin subunit gamma 2	Q13753	CS	803*	Toledo et al., 2020
Matrix-remodeling associated protein 5	Q9NR99	CS	702*	Noborn et al., 2015
Meprin A subunit alpha	Q16819	CS	631*	Nasir et al., 2016
Natriuretic peptides B	P16860	CS	41*	Toledo et al., 2020
Neurexin-1	Q9ULB1	HS	1,355	Zhang et al., 2018
Neurexin-2	Q9P2S2	HS	1,400	Zhang et al., 2018
Neurexin-3	Q9Y4C0	HS	1,315	Zhang et al., 2018
Neuropeptide W	Q8N729	CS	133*	Noborn et al., 2015
Neuroserpin	Q99574	CS	403	Noborn et al., 2015
Nidogen-2	Q14112	CS	452*, 358*	Toledo et al., 2020
Osteopontin	P10451	CS	234*, 308*	Noborn et al., 2015
Plexin domain-containing protein 1	Q8IUK5	CS	33	Noborn et al., 2015
Retinoic acid responder protein	P49788	CS	40	Nasir et al., 2016
Secretogranin-1	P05060	CS	93*, 239*	Noborn et al., 2015
Secretogranin-3	Q8WXD2	CS	37	Noborn et al., 2015
Sushi repeat-containing protein SRPX	P78539	CS	34*	Toledo et al., 2020

**B.** Novel proteoglycans identified in different model systems through glycoproteomics.				

**Protein name**	**Uniprot ID/Accession number**	**Modification^*b*^**	**Site(s)^*b*^**	**References**

***Caenorabditis elegans***				
CLE-1A protein/CPG-10	Q9U9K7	Chn	581	Noborn et al., 2018
COLlagen/CPG-11	Q22651	Chn	336*	Noborn et al., 2018
C-type lectin domain containing protein 180/CPG-12	Q19970	Chn	275, 290	Noborn et al., 2018
Dauer Up-Regulated, isoform b/CPG-13	Q7JLY2	Chn	368	Noborn et al., 2018
High Incidence of Males, isoform b/CPG-14	NCBINP_001024582.1	Chn	4,850*	Noborn et al., 2018
LiPocalin-Related protein/CPG-15	Q23163	Chn	172*	Noborn et al., 2018
FiBrilliN homolog/CPG-16	Q23587	Chn	1,079*	Noborn et al., 2018
Papilin/CPG-17	O76840	Chn	775*	Noborn et al., 2018
Protein C45E5.4/CPG-18	Q18642	Chn	38*	Noborn et al., 2018
Protein C06G1.2/CPG-19	Q17742	Chn	35*	Noborn et al., 2018
Protein K08B4.2/CPG-20	Q9TYY6	Chn	167*	Noborn et al., 2018
Protein R17.3/CPG-21	O18003	Chn	111*	Noborn et al., 2018
Protein T10E9.3/CPG-22	O01603	Chn	382, 399	Noborn et al., 2018
Protein Y39B6B.y/CPG-23	NCBI PIR T45051	Chn	313*	Noborn et al., 2018
Protein Y41D4B.26/CPG-24	Q8WSN8	Chn	194	Noborn et al., 2018
***Danio rerio***				
Uncharacterized protein si:ch73-306e8.2 isoform X1	XP_00133717.3	CS	103*	Delbaere et al., 2020
***Drosophila melanogaster***				
Protein Windpipe	Q9W266	CS	282, 335, 337	Takemura et al., 2020
***Rat INS-1 832/13, murine MIN-6 and NIT-1 cell lines***				
Chromogranin-A	P10354	HS	433	Nikpour et al., 2021
Immunoglobulin superfamily member 10	Q3V1M1	CS	679	Nikpour et al., 2021
Islet Amyloid Polypeptide	P12969	CS	28	Nikpour et al., 2021
Secretogranin-1	P35314	HS	236	Nikpour et al., 2021

**^a^*Glycosaminoglycan modifications: CS, chondroitin sulfate; HS, heparan sulfate; or Chn, chondroitin.*^*b*^Glycosaminoglycan attachment sites denote serine amino acid numbers, determined unambiguously by nLC-MS/MS or ambiguously for glycopeptides containing two or more serine residues (*). Numbers relate to the corresponding protein ID.

A similar strategy was developed for complex proteoglycans of the extracellular matrix (hyalectans) from human and bovine sources (Klein et al., 2018). In addition to CS chains, hyalectan proteoglycans are also rich *N*- and mucin-type *O*-glycosylations that decorate large parts of the proteins. The abundant glycosylation impairs effective peptide identification and reduces the overall sequence coverage achieved by mass-spectrometric based methods. By using a combination of efficient enrichment procedures for glycosylated peptides and advanced MS/MS-software analysis, the authors were able to improve the peptide sequence coverage of these complex proteoglycans and identify a number of new attachment sites, including both *N*-, mucin type *O*- and CS-glycosylations.

## Glycoproteomics: Novel CSPGs Leading to Novel Hypotheses

Although glycoproteomic approaches assist in identifying novel CSPGs, the functional relevance of a CS modification on cellular physiology is often unclear and cannot be inferred from only the CS structure or from the core protein alone. However, the identification of novel CSPGs, as well as defining novel attachment site modifications of established CSPGs, generate novel hypotheses that can be experimentally tested. Here we exemplify with a few cases of how such global or targeted glycoproteomic approaches can be combined with hypothesis-driven research to investigate structure-function relationships of CSPGs.

We recently identified the Windpipe protein as a novel CSPGs in *Drosophila melanogaster* and found that the core protein carried three separate CS chains in its extracellular domain (Takemura et al., 2020). Genetic engineering combined with morphological analyses demonstrated that Windpipe inhibited Hedgehog (Hh) signaling in a CS dependent manner. Interestingly, Windpipe-overexpression resulted in reduced wing size compared with control flies. However, the wing size was restored in genetically modified flies in which all the three CS attachment serine residues were substituted with alanine residues (thereby precluding CS modification). This study thus demonstrates a novel role of a specific CSPG in regulating Hh signaling and illustrate the potential of combining glycoproteomics with molecular and cell biological studies in a well-defined model system.

Several protein hormones are stored as amyloid-like aggregates in the secretory granules of endocrine cells (Maji et al., 2009). Most of the protein hormones require mild acidic pH and the addition of low molecular weight heparin or CS for their aggregation *in vitro*. Our finding that several prohormones carry CS chains lead us to hypothesize that the CS side chains may facilitate self-assembly of the prohormones (Noborn et al., 2015). Indeed, binding studies showed that CS promoted the assembly of the chromogranin A core protein under acidic condition, giving a possible explanation to previous observations that chromogranin A has an inherent property to assemble in the acidic milieu of secretory granules. Whether the CS side chains of other prohormones (e.g., secretogranin-1 and 3) may also influence the assembly of their respective core protein remains to be determined.

Furthermore, an affinity-enriched glycoproteomic approach was recently employed to explore the involvement of CSPGs in the pathogenesis of pregnancy-associated malaria. The disease is caused by the parasite *Plasmodium falciparum* and has potentially very severe clinical outcome for both mother and child (Toledo et al., 2020; Tomlinson et al., 2020). The parasite induces expression of the malarial protein VAR2CSA on the surface of infected erythrocytes, which enables their binding to structural variants of CS in the placental intervillous space of pregnant women (Kane and Taylor-Robinson, 2011). This interaction is dependent on the size and sulfation-type of the polysaccharide, as longer chains with higher degree of C4-*O*- sulfation of GalNAc residues display stronger binding (Sugiura et al., 2016; Ma et al., 2021). A VAR2CSA-affinity column was recently used to enrich for CSPGs, capable of binding the VAR2CSA-protein, in human placenta. The enriched fraction was then analyzed through a CS glycoproteomic workflow, showing that a collection of different core proteins, rather than a single core protein, carried CS chains that were capable of binding the VAR2CSA-protein (Toledo et al., 2020). This indicates that several different CSPGs may serve as attachment factors for malaria-infected erythrocytes, which provides new insight into the disease etiology. Notably, CS and heparan sulfate (HS) also constitute attachment factors for many enveloped and non-enveloped viruses (Olofsson and Bergström, 2005). Interestingly, recent studies show that cell surface HS act as a co-receptor to ACE2 and is essential for SARS-CoV-2 infection (Clausen et al., 2020; Mycroft-West et al., 2020). To our knowledge, however, the information of a potential HS core protein (or group of core proteins) involved in such processes is scarce (Daly et al., 2020). A glycoproteomic enrichment strategy, similar to the VAR2CSA-CS enrichment approach, may provide information on this issue.

## Proteoglycan Linkage Region Complexity

The CS linkage region has previously been perceived as a relatively uniform entity with limited structural variability. MS/MS-analysis of chondroitinase ABC-digested bikunin described a defined hexasaccharide structure with *O*-sulfations of the GalNAc residue and of the outer Gal residue (Ly et al., 2011). In addition to sulfation, other linkage region modifications have been reported, e.g., sialylation and phosphorylation (Sugahara et al., 1988; Kitagawa et al., 2008; Lu et al., 2010; Wen et al., 2014). However, the reports have been limited to only a few separate experimental systems and information on potential combinations of linkage region modifications is still scarce. To this aim, our glycoproteomic analysis of human bikunin and other proteoglycans from urine, CSF, and plasma have revealed an unexpected linkage region complexity, with different combinations of sulfation, phosphorylation and sialylation (Gomez Toledo et al., 2015; Noborn et al., 2015; Nasir et al., 2016; Nilsson et al., 2017). Furthermore, the structural variability of human bikunin was further increased by large variations in the mucin type *O*-linked glycosylation found nearby the CS site (Gomez Toledo et al., 2015). Moreover, an unexpected fucose (deoxy-hexose) modification was found on the xylose residue of the linkage region of human bikunin and decorin and on two novel human CSPGs; retinoic acid responder protein 1 and meprin A (Gomez Toledo et al., 2015; Nasir et al., 2016). This was surprising as fucosylated CS chains have previously only been described in sea cucumbers, a member of the phylum of Echinodermata (Myron et al., 2014; Dwivedi and Pomin, 2020). Notably, this fucose modification in human CS was located on xylose (and not on the CS chain), i.e., close to the protein component. This position is similar to the fucose modifications on *N*-glycans, which also may occur at the innermost GlcNAc residue linked to Asn in the consensus amino acid sequence (core fucosylation) (Plomp et al., 2017; Schneider et al., 2017; Wang and Ravetch, 2019). This kind of *N*-glycan fucosylation occurs both in invertebrate and vertebrate species and modifies the functional effects of the corresponding proteins, e.g., by regulating the inflammatory immune responses by modifying the *N-*glycans of the Fc chains of IgG1 molecules. This is of major importance when designing and introducing monoclonal antibodies for immune therapy, since the afucosylated variants show an increased antibody-dependent cellular cytotoxicity (ADCC) (Pereira et al., 2018). What functional effects the core fucosylation of xylose of CSPG may have remains to be determined.

Furthermore, we have observed a non-canonical linkage region trisaccharide (GlcA-Gal-Xyl-*O*-) on glycopeptides of bikunin from urine of healthy donors. This trisaccharide thus lacks one Gal residue of the traditional linkage region, and results in a residual pentasaccharide structure upon chondroitinase ABC digestion (Persson et al., 2019). Interestingly, mutations in the *B3GALT6* gene, which codes for galactosyltransferase II (β3GalT6) is associated with Ehlers-Danlos–like syndromes, characterized by a spectrum of skeletal and connective tissue “linkeropathy” disorders (Malfait et al., 2013). The β3GalT6 enzyme is responsible for adding the second Gal residue in the GAG linkage region and mutations in the gene results in reduced GAG biosynthesis and appearance of the trisaccharide linkage region in a zebrafish *b3galt6* knock-out model (Delbaere et al., 2020). Whether patients with *B3GALT6* mutations also have increased levels of the non-canonical trisaccharide linkage region of bikunin in their urine remains to be determined. Taken together, at least 14 different variants of the bikunin linkage region (including canonical and non-canonical sequences) have now been described, thus demonstrating a much greater variability than previously appreciated (Gomez Toledo et al., 2015; Noborn et al., 2015; Nilsson et al., 2017; Persson et al., 2019). Xylose phosphorylation has been shown to function as a molecular switch to regulate the proteoglycan biosynthesis (Wen et al., 2014). The removal of xylose phosphorylation by 2-phosphoxylose phosphatase is considered a prerequisite for the polymerization of the linkage regions into longer chains (Koike et al., 2014). However, the influence of the other linkage modifications (or the combinations thereof) on the downstream GAG biosynthesis is yet unknown. Nevertheless, knowledge of linkage region complexity provides a theoretical framework for future functional-structural studies of CS biosynthesis. Moreover, analysis of extended site-specific CS chains, in theory achieved by using partial chondroitinase ABC digestion, may assist in further understanding of how these modifications affect the biosynthesis and thus the final structures of CS. In addition, this may provide information on how certain core protein determinants (e.g., amino acid sequences) may influence the CS structures. The concept of core protein specific structures have been difficult to address since most established proteoglycan workflows involve the separation of the CS polysaccharides and the core proteins. Ideally, site-specific analysis of full-length CS structures is desirable, as this type of information is likely required to fully explore the structural-functional basis of CSPGs.

Further development of the glycoproteomic concept has enabled site-specific characterization of both CS and HS sites (Noborn et al., 2016). To this aim, perlecan (also known as basement membrane specific proteoglycan) was chosen as a model proteoglycan since it is known to be substituted with both CS and HS chains (Iozzo, 2005; Noborn et al., 2015). A trypsin-digested perlecan sample, derived from Engelbreth-Holm-Swarm mouse sarcoma, was enriched on a SAX column and digested with heparinase or chondroitinase ABC, generating residual tetrasaccharide and hexasaccharide structures still attached to the peptide. This allowed for the identification of a glycopeptide derived from the N-terminal domain of perlecan, which encompassed the three previously known HS sites (Iozzo and Sanderson, 2011). In addition, the combination of chondroitinase and heparinase digestion revealed a “hybrid site” in the C-terminal domain, carrying either an HS or a CS chain (Noborn et al., 2016). This was surprising since it demonstrated that a single GAG attachment site is capable of carrying either HS or CS chains, thus, revealing a less appreciated level of proteoglycan heterogeneity. The identification of such “hybrid sites” is likely to be biologically important as HS and CS often display opposite effects on cellular physiology (Coles et al., 2011; Doody et al., 2015). Furthermore, similar glycoproteomic protocols have been used to identify novel HSPGs, and to investigate the functional role of HSPGs in neuronal synapse formation (Zhang et al., 2018; Montoliu-Gaya et al., 2021; Nikpour et al., 2021).

## Expanding the Proteoglycan Glycoproteome — But How Far?

Glycosylation is one of the most ubiquitous forms of post-translational modification and is expected to be present on more than half of all mammalian proteins (Apweiler et al., 1999; Schjoldager et al., 2020). However, despite its high abundance, GAG modifications are considered to occur only on a very minor proportion of the 20,000–25,000 proteins encoded by the human genome. Although glycoproteomic studies of various sample matrices have significantly increased our structural knowledge of proteoglycans, fewer than 80 core proteins carrying CS have so far been identified in humans (Noborn et al., 2015; Nasir et al., 2016; Toledo et al., 2020). A list of all CSPGs known to date in humans was recently published by Toledo et al. (2020). The number of human core proteins carrying HS are even less, and so far only a little more than 20 HSPGs have been established (Zhang et al., 2018; Nikpour et al., 2021). Moreover, while proteoglycans in vertebrates have been the focus of several structural studies, the information about proteoglycans in invertebrates is scarce and the reports are restricted to only a few species (Noborn and Larson, 2021). Thus, only a limited number of CSPGs have been identified in *Drosophila melanogaster*, which is surprising since it is one of the most studied invertebrates (Momota et al., 2011; Zhu et al., 2019; Takemura et al., 2020). Future studies employing glycoproteomic approaches will likely identify novel CSPGs of invertebrates and further expand our knowledge of the proteoglycan glycoproteome in different species.

**FIGURE 1:**
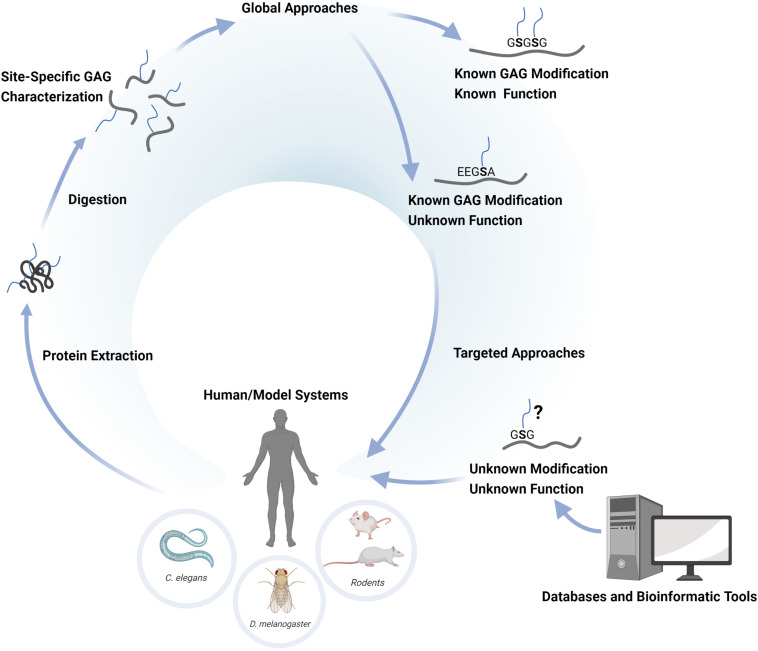
Schematic illustration of the basic concepts concerning glycoproteomic identification of proteoglycans. The identification is achieved by the extraction of proteoglycans from human tissues or from various experimental systems (*C. elegans*, *D. melanogaster*, etc.), followed by the digestion with trypsin and chondroitinase ABC to enrich and generate defined CS-glycopeptides. The glycopeptides are then analyzed by nLC-MS/MS and the data is evaluated through glycopeptide search algorithms. This approach provides a global site-specific characterization of the CSPGs present in the sample system of interest. The identified CSPGs may be previously known CSPGs with known functions, or novel CSPGs for which the functions are yet unknown. The use of targeted approaches (e.g. molecular engineering) may assist in exploring the potential function of novel CSPGs in the sample system of interest. Bioinformatic analysis of the proteome sequences of a given model system may provide information of potential GAG-attachment sites. An assumed GAG-site may thus be verified in vivo using targeted glycoproteomic approaches and functional read-outs.

The enzymatic transfer of a Xyl residue to certain Ser residues in the proteoglycan core protein initiates GAG biosynthesis (Briggs and Hohenester, 2018). The Xyl-modified Ser residue is typically followed by a Gly residue (-SG-) and is associated with a cluster of acidic residues in close proximity, indicating that certain motifs of the amino acid sequence influence the initiation process (Esko and Zhang, 1996). Such motifs may assist in the prediction of potential GAG sites of core proteins and provides insights into the potential space of the GAG glycoproteome. When we recently mapped the chondroitin (Chn) glycoproteome in *C. elegans*, this resulted in the identification of 15 novel core proteins in addition to the 9 previously established core proteins (Olson et al., 2006; Noborn et al., 2018). Bioinformatic analysis of protein sequences in the regions of the glycosylated Ser residues showed a highly stringent attachment motif E/D-G/A-S-G. A search in the Swiss-Prot database using this motif retrieved 19 additional potential CPGs, indicating that possibly additional CPGs are yet to be found in the nematode (Noborn et al., 2018). However, it is unclear why these assumed CPGs escaped detection by the glycoproteomic analysis. This may simply relate to the sensitivity of the assay and that the glycopeptides corresponding to these potential CPGs were below the detection threshold. On the other hand, it may also suggest that some motifs are only occasionally occupied, or substituted with HS instead of Chn. Nevertheless, this demonstrates the ambiguity of using only attachment motifs as a strategy to identify proteoglycans, and that glycoproteomic analysis is needed to determine the GAG glycoproteome in any given sample system. The basic strategies for proteoglycan identification and the different levels of structural- and functional understanding of their roles in any biological system is schematically illustrated in Figure 1.

The prohormone chromogranin-A (CgA) is one of the main proteins in secretory granules of endocrine cells and the precursor for several bioactive peptides (Kim et al., 2001). CgA has previously been established as a CSPG and carries a CS chain in the C-terminal end (Ser-424) of the human core protein (Gowda et al., 1990; Noborn et al., 2015). Our recent glycoproteomic analysis of cultured insulin-secreting cells, demonstrate that CgA in rodents, but not in humans, also carries HS at the same C-terminal site, making this another “hybrid site” (Noborn et al., 2016; Nikpour et al., 2021). Although the glycosite is highly conserved between species (Noborn et al., 2015), this highlights the importance of cell-targeted mapping and that GAG modifications found in one cell type or tissue or species may not simply be inferred to another sample system. These findings also support the view that the GAG glycoproteome should not be regarded as a static entity, but rather a dynamic system that changes with the cellular physiology. A key issue for the future is to comprehend the biological factors that eventually will govern the expansion of the GAG glycoproteome.

## Discussion

As with any research area, the characterization of any particular biomolecule is critical for the understanding of its role in biological and pathological processes. The identification and structural characterization of proteoglycans is no exception. According to our current understanding of CSPGs, these are often regarded in a functional context, in which any given proteoglycan regulate a specific function in cellular pathophysiology. For example, decorin, a small leucine-rich extracellular matrix proteoglycan, is well-studied for its role as a suppressor of tumor cell growth (Neill et al., 2016; Appunni et al., 2019). Decorin binds and antagonizes various receptor tyrosine kinases, thereby inhibiting downstream oncogenic signaling and reducing tumor growth (Reszegi et al., 2020; Hu X. et al., 2021). Apart from their involvement in cancer, CSPGs are also in focus of other research areas, including neurogenesis and spinal cord injury (Maeda, 2015). Following damage to the central nervous system, axons fail to regenerate due to the formation of a glial scar, which is composed of extracellular matrix components including CSPGs (Hussein et al., 2020). The production of several CSPG family members is differentially regulated in the glial scar as neurocan, brevican and versican are increased, while aggrecan is reduced (Mukhamedshina et al., 2019). How the individual CSPGs contribute to the pathogenesis is yet unclear, but their relevance is illustrated by the interest of using chondroitinase ABC as a therapeutic agent (Bradbury et al., 2002; Hu et al., 2018; Hu J. et al., 2021). Although detailed structural-functional understanding of PGs many times remains unknown, the examples given in this review point to essential roles of CSPGs in various pathophysiological conditions and demonstrate the significant progress that has been made in the field in recent years.

The recent advances in glycoproteomics have enabled the identification of several novel CS core proteins in humans and in various animal models. With such techniques, additional CS core proteins and novel GAG modifications will likely be discovered with no yet assigned function/s. Thus, one should consider whether “a proteoglycan” should be defined mainly on the basis of an assigned function or on structural evidence of its existence. Importantly, glycoproteomic strategies have the potential of finding many novel proteoglycans and provide global structural information that may contribute to our conceptual understanding of the complex family of proteoglycans. However, viewing proteoglycans from a “structure only” perspective will meet its limitations. The development of even more advanced glycoproteomics strategies will presumably identify proteoglycans at low abundances and/or at low levels of occupancy. This suggests that caution is needed in the interpretation of the data as minute amounts of any given proteoglycan may have little or no relevance for our understanding of any given biological system. Therefore, using glycoproteomics in a clearly defined biological or pathological context will likely be a necessary future strategy, since it will set constraints on the interpretation of the structural information derived. This will likely assist in determining which proteoglycans are relevant for understanding the underlying biological mechanisms in any given system. Nonetheless, glycoproteomic strategies will surely assist in further expanding the knowledge of proteoglycan core proteins and their functions. In the perspective of the present pandemic of COVID-19, the importance of proteoglycans as attachment factors/receptors or co-receptors of pathogenic viruses cannot be underestimated and detailed structural information of the host GAGs and their core protein structures will most likely be warranted to understand the molecular details of virus infections as well as to design novel anti-viral drugs.

## Author Contributions

FN and GL did the writing and provided the main concepts discussed in this mini review. MN, AP, and JN assisted in the organization and editing of the publication and contributed to the table and the figure. All authors contributed to the article and approved the submitted version.

## Conflict of Interest

The authors declare that the research was conducted in the absence of any commercial or financial relationships that could be construed as a potential conflict of interest.

## Publisher’s Note

All claims expressed in this article are solely those of the authors and do not necessarily represent those of their affiliated organizations, or those of the publisher, the editors and the reviewers. Any product that may be evaluated in this article, or claim that may be made by its manufacturer, is not guaranteed or endorsed by the publisher.
